# The world of trauma working together

**DOI:** 10.1186/1749-7922-7-S1-S2

**Published:** 2012-08-22

**Authors:** Sandro Rizoli, Ari Leppäniemi

**Affiliations:** 1 University of Toronto, Canadá; 2 University of Helsinki, Finland

## 

The World Trauma Congress is a success even before its official opening next August 22^nd^. For the first time 10 national and multinational Trauma Associations plus health professionals from more than 40 countries will get together to confer on the science and status of trauma in the world in the 21^st^ century.

Trauma is medicine practiced by teams/groups of health professionals. The field of trauma extends from injury prevention to trauma systems, from pre-hospital care to rehabilitation with lots in between including several hospital-based professionals (i.e. nurses, surgeons, anesthetists, intensivists, technologists, physiotherapists and others). As trauma evolves and the necessity to become more structured and organized is recognized by countries across the world, the importance of local Trauma Associations has been rediscovered. In turn, as the local/national Trauma Associations become stronger and more relevant to their communities and countries, they also see the value of participating in multinational Associations. This process has resulted in the resurgence of the Panamerican Trauma Society in the Americas, the creation in Europe of the European Society for Trauma and Emergency Surgery (ESTES) and the proliferation of international education programs by the American College of Surgeons Committee on Trauma. Now in 2012, these national and multinational associations will gather under the umbrella of the first World Trauma Congress.

The goal of having a truly World Trauma Congress that represents all the aspirations of all Trauma Associations of the world will only be partially fulfilled in August. But the meeting shows that the trauma world is capable of getting together, sharing knowledge and working together to improve trauma care everywhere.

One of the highlights of the World Trauma Congress is 2 separate scientific Journal supplements. All participants of the World Trauma Congress were invited to submit full manuscripts to these supplements and 11 were selected by peer-reviewed process for the present supplement. These manuscripts address many of the most important topics in trauma in the world today such as deaths due to motorcycle crashes. While rich countries appear primarily concerned about fossil fuel cost to move their large fleet and its ecological impact, developing nations are experiencing epidemic proportions of death related to the growing numbers of small and economical motorcycles [1]. The cost to purchase and maintain a motorcycle is low, which makes it attractive to developing and poor nations where citizens also lack resources to purchase safety equipment and the state does not provide adequate roads or traffic law enforcement. The final result is an alarming and continuously growing number of deaths associated to motorcycle in Latin America and Asia, where full hospital wards care for hundreds of invalid survivors. Motorcycle crash was also the mechanism of injury most frequently described in another manuscript on the non-operative management of high grade (grade IV) hepatic also included in this supplement [2].

The background for the first 2 studies contrasts with that of two other manuscripts that analyze the utility of robots and telemedicine in medical education and exchange of trauma knowledge [3,4]. Education is also the focus of another study from Curitiba, Brazil, investigating how many hours are necessary for medical students to become proficient in some Emergency Department tasks [5]. The rational for the study is the fact that in developing countries, recently graduated physicians with deficient training in Emergency Medicine, are the ones staffing most Emergency Departments of the country. This reality contrasts with that of nations where Emergency Medicine is a medical specialty requiring 3 to 5 years of post-graduate (residency) training.

This supplement also selected high caliber experimental research and novel diagnostic methods and therapies. Dr Rezende [6] reports an exceptional experimental study on tissue perfusion during “permissive hypotension” resuscitation. This work was awarded the best paper at the 2011 Eastern Association for the Surgery of Trauma annual meeting. Another interesting manuscript reports on the role of alcohol and sepsis on the tensile strength of bowel anastomosis [7]. On novel diagnostic methods and therapies, Dr Sankarankutty reviewed the literature on the possible role of thromboelastometry [8] while another study reports on the lack of utility for recombinant factor VIIa in trauma [9].

Finally two manuscripts focus on the “growing pains” experienced by developing countries as they try to implement complex and costly trauma systems. Dr Gonsaga and collaborators [10] compared two pre-hospital ambulance transportation systems: one newly created and another functioning for years, both public and free (funded by government) and serving the same population. While this analysis demonstrates the growing governmental investments in pre-hospital care, it also reveals inefficiencies of the system (i.e. service duplication). The final manuscript brings hope. Dr Fraga and collaborators [11] started their manuscript with the gloomy hypothesis that the ending of the Trauma Surgery residency in Brazil in 2003 would be followed by a reduction in the number of manuscripts published in trauma. The authors however, found the contrary. Scientific production in Brazil, measured by publications in trauma grew continuously before and after the end of the residency program. This study shows the resiliency and determination of the academic surgeons in Brazil and the benefits of having a strong National Trauma Association such as the Brazilian SBAIT (Society for the Integral Care of the Traumatized).

It is with this hope that we see the World Trauma Congress. Despite many barriers, national and multinational Trauma Associations from around the world are getting stronger, are increasing their participation in health policies and are becoming more influent. And these associations and their members are also recognizing the importance of collaborating and they will get together in August 2012 with the goal of ending trauma in the world. A goal we are proud to be part of.

## Competing interests

The authors declare that they have no competing interests.
